# Effects of Auditory Distraction on Face Memory

**DOI:** 10.1038/s41598-019-46641-7

**Published:** 2019-07-15

**Authors:** Raoul Bell, Laura Mieth, Jan Philipp Röer, Axel Buchner

**Affiliations:** 1 Department of Experimental Psychology, Heinrich Heine University Düsseldorf, Düsseldorf, Germany; 2 Department of Psychology and Psychotherapy, Witten/Herdecke University, Witten, Germany

**Keywords:** Short-term memory, Working memory, Human behaviour

## Abstract

Effects of auditory distraction by task-irrelevant background speech on the immediate serial recall of verbal material are well established. Less is known about the influence of background speech on memory for visual configural information. A recent study demonstrated that face learning is disrupted by joyful music relative to soothing violin music and quiet. This pattern is parallel to findings in the serial-recall paradigm showing that auditory distraction is primarily caused by auditory changes. Here we connect these two streams of research by testing whether face learning is impaired by irrelevant speech. Participants learned faces either in quiet or while ignoring auditory changing-state sequences (sentential speech) or steady-state sequences (word repetitions). Face recognition was impaired by irrelevant speech relative to quiet. Furthermore, changing-state speech disrupted performance more than steady-state speech. The results were replicated in a second study using reversed speech, suggesting that the disruptive potential of the background speech does not depend on its semantic content. These findings thus demonstrate robust effects of auditory distraction on face learning. Theoretical explanations and applied implications are discussed.

## Introduction

People usually prefer a quiet environment to study because they are concerned that learning may be impaired by background sounds. Research has indeed confirmed that important working-memory functions such as maintenance^1^, updating^2^, problem solving^3^, and binding^4^ suffer when task-irrelevant speech has to be ignored. As yet, research has mainly focused on the effects of auditory distraction on short-term memory for verbal information (e.g.^5^). Here, we test whether the disruptive effects of background speech generalize to face learning. This is relevant for the evaluation of theories about auditory distraction but also for applied contexts such as eyewitness testimonies^6,7^.

The distracting effect of task-irrelevant background sounds relative to quiet is often referred to as *irrelevant sound effect*^8–12^. The disruption of working-memory processes is mainly determined by the degree to which the auditory signal is changing abruptly. One of the key signatures of auditory distraction is the *changing-state effect*, which refers to the observation that auditory distractor sequences that contain many different auditory objects (such as words or tones) disrupt cognitive performance more than continuous or repetitive sounds^9,13,14^. Most research focuses on the serial-recall paradigm in which short lists of verbal items (words, digits, or consonants) have to be recalled in the correct order, either immediately after presentation or after a short retention interval^5^. The typical finding is that changing-state sequences that are composed of different one-syllable words^15–17^ or sentential speech^1,18,19^ disrupt serial recall more than so-called steady-state sequences that consist of regular repetitions of the same distractor word. The changing-state effect is also thought to be responsible for the surprising finding that multiple voices disrupt performance less than a single voice because when listening to multiple voices the changes in each speech signal are masked by the other voices so that the resulting signal is smoothed out^20^. The disruptive effect of irrelevant speech on working-memory processes cannot be solely attributed to its lexical, syntactic, or semantic properties as speech has a strong impact on performance even when it is incomprehensible because it is reversed^19,21–23^. Compared to changing speech, steady-state speech consisting of repeated speech utterances such as consonants or one-syllable words cause less disruption^9,14,15^. Until recently, it was widely believed that steady-state stimuli cause no reliable disruption at all (e.g.^11^). However, the disruptive effect of steady-state speech is simply smaller than that of changing-state speech so that it is not always detected in statistical tests^24^.

Auditory distraction is not limited to speech^19,25–30^. Klatte, Kilcher, and Hellbrück^31^ showed that a musical piece played *staccato* disrupted performance whereas the same piece played *legato* did not disrupt performance relative to a quiet control condition (see also^27^). Furthermore, sequences consisting of glissandi (continuous pitch glides) did not cause distraction compared to a quiet control condition. By contrast, the same auditory sequences disrupt performance when the glissandi are interrupted by periods of silence, rendering the sequence into distinct auditory objects with abrupt onsets^32^. Furthermore, changing tones disrupt immediate memory more than repeated tones^13^. The disruptive effect of irrelevant non-speech sound is thus characterized by a changing-state effect, just as that of irrelevant speech^13,14^.

While the effect of task-irrelevant sounds on working-memory processes is comparatively well understood, less is known about how auditory distractors affect the processing of complex visual stimuli (cf.^33^). In particular, the question of whether face learning is affected by task-irrelevant background speech is interesting from a theoretical as well as from an applied perspective. In forensic settings, for instance, it is important to know whether the identification of a perpetrator face is affected by background noise^6,7^.

The present study was inspired by a recent study of Proverbio *et al*.^34^ who examined how background music affects face learning. In their experiment 300 faces were presented while participants listened to joyful music, touching music, the sound of rain, or quiet. Face recognition was better when participants had listened to touching music at encoding than when they had listened to joyful music or rain. Proverbio *et al*. discussed their results in light of an arousal-and-mood hypothesis suggesting that the “binding of facial properties with auditory and emotionally charged information (music) … may result in deeper memory encoding” (p. 1). However, they also mentioned several aspects of their results that are more consistent with an interpretation in terms of auditory distraction. First, Proverbio *et al*.’s measures of emotional arousal (blood pressure and heart rate) were numerically higher for both joyful and touching music in comparison to rain and quiet and thus did not follow the same pattern as face recognition which was disrupted by joyful but not by touching music. Second, there was no beneficial effect of touching music on face recognition compared to the quiet control condition. Instead, face recognition was impaired when the faces were encoded while listening to joyful music or rain in comparison to quiet. This raises a theoretically interesting question: Could these effects be due to auditory distraction?

**Figure 1:**
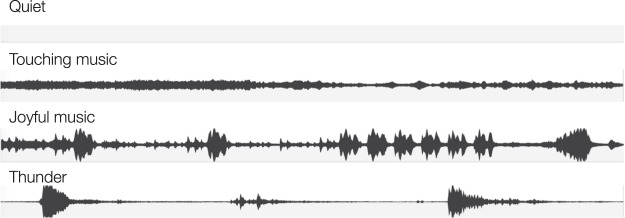
Examples for the stimulus material used in the study of Proverbio *et al*.^34^: A quiet control condition, emotionally touching music (Arvo Pärt: Cantus in memoriam Benjamin Britten), joyful music (Paul Hindemith: Kammermusik No. 1, 1st movement), sound of rain with bursts of thunder (available at https://youtu.be/WvRv-243Cmk).

The finding that joyful music caused a large amount of interference while the emotionally touching music did not affect performance relative to quiet is consistent with a changing-state effect. In the study of Proverbio *et al*.^34^, the joyful music contained a large number of changes in quick succession (see Fig. 1) while the emotionally touching music consisted almost entirely of glissandi. The finding that the joyful music disrupted performance more than the emotionally touching music can be explained with the general principle that auditory sequences with many abrupt changes disrupt memory more than sequences containing no such changes. At first glance, it seems surprising that the sound of rain should interfere with memory because rain usually causes a regular sound pattern that does not contain any abrupt changes. However, in the sound file used by Proverbio *et al*., this regular pattern was interrupted by sudden bursts of thunder (see Fig. 1). Therefore, the disruption of memory in this condition in comparison to the quiet control condition is in line with the common finding that sudden deviations from regular patterns capture attention and cause distraction^2,35–39^.

The results of Proverbio *et al*.^34^ may thus provide evidence for an effect of auditory distraction on face learning. This finding is important because as yet evidence for a disruption of face processing by irrelevant sound is not well documented, due to a lack of systematic research on the influence of auditory distraction on visual memory (cf.^33^). There are, however, a few pioneering studies^6,7^ that provide preliminary evidence for an irrelevant speech effect on face processing. In these studies, eyewitnesses were required to create a facial composite of a perpetrator face with the help of a computer program. The quality of the facial composites was affected by background speech. In the first study^6^, interference depended on whether the content of the irrelevant speech was incongruent with the target face. The verbal description of another face with different facial features caused a reduced likeness of the created facial composites while the congruent description of the face of the perpetrator did not affect the quality of the facial composites. In a follow-up study^7^, irrelevant speech, per se, did not negatively influence the quality of the facial composites and the identification of the perpetrator face in a photo lineup. Meaningful halfalogues (overhearing one side of a cell-phone conversation), by contrast, disrupted both the quality of the facial composites as well as the identification of the perpetrator face in the lineup. This finding was explained by attentional diversion. Halfalogues contain less information than dialogues but are more difficult to predict. The unpredictability of the auditory input thus seems to be a more important determinant of auditory distraction than the amount of information that has to be ignored.

While first evidence thus suggests that special properties of speech can distort the memory representations of faces^6^ and cause attentional diversion^7^, it is as yet unclear whether face learning is affected by the mere presence of irrelevant speech. In fact, irrelevant speech, per se, did not seem to have a reliable effect on face processing relative to a quiet control condition in both aforementioned studies^6,7^. However, Marsh *et al*.^7^ refer to an unpublished study providing evidence in favor of a changing-state effect on face learning. Lineup identification accuracy was worse when a changing-state sequence (made up of different letters) had to be ignored during the encoding of the perpetrator face than when a steady-state sequence (made up of a repeated letter) had to be ignored. While the two studies providing evidence against an irrelevant speech effect^6,7^ focused on the *incidental* learning of faces, the unpublished study providing evidence in favor of such an effect focused on the *intentional* learning of faces, just as the study of Proverbio *et al*.^34^. This suggests that intentional face learning may indeed be affected by irrelevant speech. However, given that this assumption as yet relies only on a single unpublished study, more research is needed to determine the robustness of the irrelevant speech effect on intentional face learning. The present study focuses on the distraction by sentential speech. As the central medium of human communication, sentential speech is a ubiquitous part of many acoustic environments. It is therefore important to understand its disruptive power on memory for theoretical as well as for applied reasons.

The present study aims at testing whether intentional face learning is impaired by irrelevant speech. The study was modeled after that of Proverbio *et al*.^34^ in that it involved an intentional learning procedure but speech instead of music was played during face presentation. As in our previous studies^38,40–42^, we contrasted changing-state sequences consisting of sentential speech with so-called steady-state sequences in which the same word was repeatedly presented. To arrive at clear conclusions about the vulnerability of face processing to auditory distraction, we averaged over a large number of faces and used large sample sizes of over 100 participants in each study to decrease the impact of unsystematic variance and to obtain sufficient statistical power.

## Study 1

### Method

#### Participants

The final sample consisted of 103 participants who were recruited at Heinrich Heine University Düsseldorf (74 female; mean age of 23, *SD* = 5). Three datasets had to be removed because three students had participated twice (the data sets from their first participation were retained).

#### Materials

For each participant, color photographs (256 × 384 pixels) of 60 female and 60 male faces were randomly drawn from a pool of 100 adult female and 100 adult male faces from the FERET database^43^. Each participant saw a different, randomly selected set of faces. For the encoding phase, 15 sequences of four faces were created by randomly drawing faces from the pool (without replacement) with the restriction that each sequence contained two male and two female faces in random order. Each face was shown in frontal view at the center of the computer screen. Sixty other faces (30 female, 30 male) were randomly drawn from the same pool to serve as new faces in the old-new recognition test.

For the auditory distractor sequences, we used the same stimulus material as in the study of Röer *et al*.^40^. In the changing-state condition, sentential speech was used (e.g.,“Peel and quarter the onions and slice them into thin pieces, then add the tomatoes, then simmer at medium heat”; translated from German). The sentences were taken from eight different categories (weather forecast, prose text, cooking recipe, scientific textbook, poem, operating manual, road message, aphorism). For each steady-state sequence, a monosyllabic word (from the sentence) was randomly selected and repeated 18 times. This corresponded to the mean number of words in the changing-state sequences. The auditory sequences were spoken by a male voice and lasted 8 s each. With this material, a robust changing-state effect on serial recall has been obtained in several previous studies^30,38,40–42,44^. The sounds were played binaurally at about 65 dB (A) using headphones with high-insulation hearing protection covers that were plugged directly into the Apple iMac computer which controlled the experiment.

#### Procedure

A standalone application built with LiveCode [https://livecode.com] controlled the experiment. Participants received standard written instructions on the computer screen informing them that they should concentrate only on the faces which have to be recognized later and ignore the speech which is completely irrelevant for the task.

For each participant, the sequences of faces were randomly assigned to the three conditions (quiet, steady state, changing state). Participants started each trial by pressing a “continue” button. This initiated the presentation of the auditory distractor sequences. After 1200 ms, the first face was shown. The four faces of a sequence were presented one after another, each for 700 ms with a 1200 ms inter-stimulus interval. Immediately after the offset of the last face, the “continue” button appeared with which participants started the next trial. Pilot testing had shown that it was necessary to repeat the presentation of the faces to obtain above-floor memory performance when presenting a large number of faces in quick succession, as in the study of Proverbio *et al*.^34^. Therefore, all of the 15 face sequences (each consisting of 4 faces) were presented three times, each time in a new, randomly determined order. All 60 faces were shown before being repeated. To prevent habituation to the auditory sequences (cf.^19^), the faces assigned to the distractor conditions were accompanied by different changing-state or steady-state sequences each time they were presented. All encoding trials were completed prior to the test phase.

In the test phase, the 60 old faces (20 of each auditory condition) were intermixed with 60 new faces. The participants’ task was to classify each face as old or new. For each participant, the faces were presented in random order. The order of the faces at test was thus entirely unrelated to the order with which the faces were presented at encoding. In each trial of the recognition test, a face was presented in the middle of the screen along with the headline “Was this face old or new?”, and two buttons (labeled “old” and “new”). Upon clicking one of the buttons, a “continue” button appeared. Upon clicking this button, the next face was shown.

Written informed consent was obtained from all participants. Ethical approval had been obtained from the ethics committee of the Faculty of Mathematics and Natural Sciences at Heinrich Heine University Düsseldorf. The study involved standard procedures in accordance with the ethical guidelines published by the American Psychological Association and the Declaration of Helsinki. The data are available at https://osf.io/ksqx9/.

#### Results

**Figure 2:**
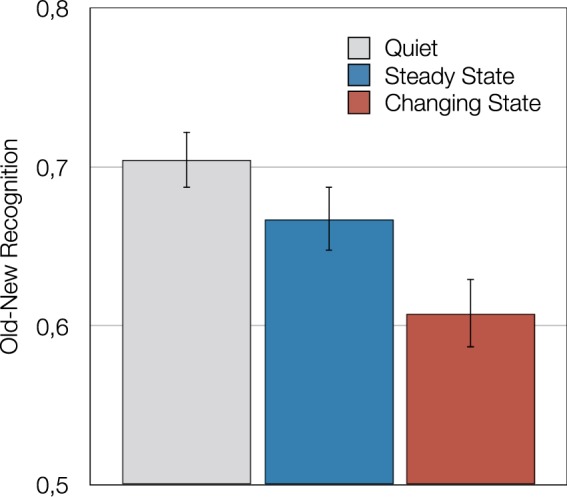
Old-new face recognition in terms of the sensitivity measure of the two-high threshold model^45^—measured by hit rate minus false alarm rate—as a function of distractor condition (quiet, steady state, changing state) in Study 1. All distractors were played in forward direction. The error bars represent the standard errors of the means.

Figure 2 shows old-new recognition as a function of distractor condition in terms of *P*_r_. The sensitivity measure of the two-high threshold model was chosen because it has been positively evaluated in validation studies (e.g.^45^) and avoids the problem of undefined values that comes with using *d*′. The measure is often referred to as “corrected hit rate” because it is calculated by subtracting the false alarm rate from the hit rate. There was a main effect of auditory distraction on face recognition, *F*(2,101) = 20.38, *p* < 0.001, η_p_^2^ = 0.29. Orthogonal contrasts showed that performance in the quiet control condition was better than performance in the two distractor conditions, *F*(1,102) = 33.12, *p* < 0.001, η_p_^2^ = 0.25, which provides evidence of an irrelevant speech effect. Furthermore, changing-state sequences disrupted performance more than steady-state sequences, *F*(1,102) = 15.93, *p* < 0.001, η_p_^2^ = 0.14, which represents evidence of a changing-state effect. Note that an analysis of *d*′ leads to the same statistical conclusions.

#### Discussion

The results of Study 1 confirm that intentional face learning is disrupted by distractor speech. Furthermore, the pattern of findings is consistent with a changing-state effect. Changing-state sequences consisting of sentential speech disrupted face learning more than steady-state sequences consisting of word repetitions. According to the changing-state explanation, the increased distraction caused by sentential speech in comparison to word repetitions is primarily due to auditory changes in the distractor material.

However, the changing-state sequences did not only comprise more changes, they also contained more semantic information than the steady-state sequences. In principle, the data pattern of Study 1 could be explained by assuming that the semantic processing of the sentences diverted resources away from the face-learning task. This semantic-interference hypothesis has the disadvantage that it cannot account equally well for the disruption of face learning by sequences of non-speech sounds observed in the study of Proverbio *et al*.^34^. Furthermore, the literature on auditory distraction suggests that semantic content of the irrelevant speech has no influence on distraction unless the primary task strongly depends on semantic processing^46^ or unless the speech captures attention because it is emotionally salient^47^ or violates expectations^48^. Furthermore, it is well established that the semantic processing of speech is not a necessary condition for obtaining auditory distraction effects on memory as such effects have often been observed with non-speech sounds such as instrumental music^14,49^ as well.

Nevertheless, to substantiate that the semantic processing of distractor speech is not a necessary condition for obtaining a disruptive effect on face learning, it is important to replicate the changing-state effect with distractor material that does not lend itself equally well to semantic processing as forward speech. Within the literature on auditory distraction, this is typically done by using reversed distractor speech that has been shown to disrupt working-memory processing in several studies^19,21–23^. In Study 2, we used the same stimulus material as in Study 1 but the distractor sequences were played in reverse. Acoustically, reversed speech has similar properties (i.e., contains a similar amount of acoustic changes) as forward speech. The changing-state hypothesis therefore implies that both the irrelevant speech effect and the changing-state effect should be observed with reversed speech as well.

## Study 2

### Method

#### Participants

The sample consisted of 106 participants who were recruited at Heinrich Heine University Düsseldorf (70 female; mean age of 24, *SD* = 4).

#### Materials and procedure

Materials and procedure were identical to those of Study 1 with the following exceptions. The same sound files were used as in Study 1, but the sound files for the changing-state sequences as well as the sound files for the steady-state sequences were reversed. For each participant, one steady-state sequence was randomly selected. This sequence was played in all steady-state trials throughout the experiment.

#### Results

**Figure 3:**
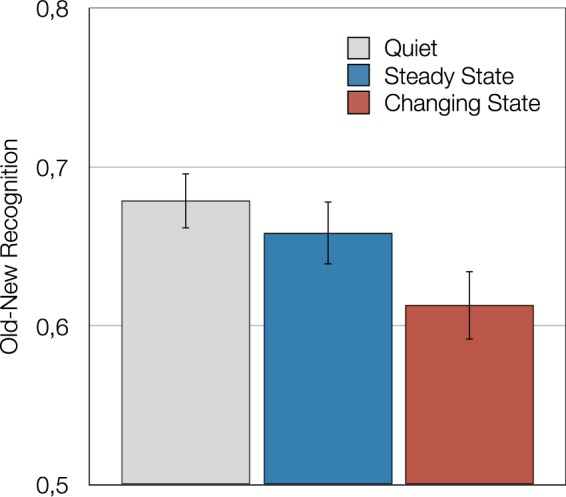
Old-new face recognition in terms of the sensitivity measure of the two-high threshold model^45^—measured by hit rate minus false alarm rate—as a function of distractor condition (quiet, steady state, changing state) in Study 2. All distractors were played in backward direction. The error bars represent the standard errors of the means.

Figure 3 shows face recognition as a function of distractor condition in terms of *P*_r_. There was a main effect of auditory distraction on face recognition, *F*(2,104) = 12.13, *p* < 0.001, η_p_^2^ = 0.19. Orthogonal contrasts showed that performance in the quiet control condition was superior to that in the two distractor conditions, *F*(1,105) = 14.26, *p* < 0.001, η_p_^2^ = 0.12, which provides evidence of an irrelevant speech effect. As in Study 1, changing-state sequences disrupted performance more than steady-state sequences, *F*(1,105) = 10.06, *p* = 0.002, η_p_^2^ = 0.09, which represents evidence of a changing-state effect. As in Study 1, an analysis of *d*′ leads to the same statistical conclusions.

#### Discussion

In Study 2, the effect of auditory distraction on intentional face learning was successfully replicated. As in Study 1, face recognition was better when the faces were encoded in quiet than when distractor speech had to be ignored. This finding generalizes the irrelevant speech effect to face learning. Furthermore, there was also a significant changing-state effect in that sentential speech disrupted face learning more than the repeated presentation of the same auditory distractor word. These results were obtained even though the speech was reversed suggesting auditory distraction effects on face learning do not depend on semantic processing, consistent with the finding that music disrupted face learning as well^34^.

This finding is parallel to previously observed effects of auditory distraction on working memory. Semantic processing of comprehensible speech is no necessary precondition for observing effects of auditory distraction on working-memory processes as disruptive effects are commonly observed with reversed speech as well^19,21–23^. Furthermore, effects of auditory distraction on working-memory processing are not restricted to speech, but are robustly observed with non-speech sounds such as music^19,25–30^ or environmental sounds^49,50^.

## General Discussion

Two studies consistently show that intentional face learning is disrupted by irrelevant speech. The disruptive effect of changing-state sequences consisting of sentential speech was stronger than that of steady-state sequences consisting of the repeated presentation of a single distractor word. The results thus suggest that auditory distraction by changing-state sounds generalizes to face learning. Here, we were able to demonstrate this with changing-state sequences whose disruptive effects on serial recall have been established in several previous studies^38,40–42^. The changing-state effect was obtained regardless of whether the speech was reversed (Study 2) or not (Study 1), suggesting that the disruptive effect of speech on face learning is not restricted to meaningful speech. A changing-state effect could also be responsible for the disruptive effect of joyful music on face learning observed in the study of Proverbio *et al*.^34^. They observed that joyful music with many abrupt changes disrupted face learning more than touching violin music with smooth transitions from one tone to another. This is parallel to the disruptive effects of staccato music on serial recall that have been attributed to a changing-state effect^27,31^.

How can the robust disruption of face learning by auditory distractors be explained? Given that in the present study speech was only played at encoding but not at retrieval, the retrieval context differs from the encoding context. At first glance, *context-dependent memory*^51^ seems to be a candidate explanation for the effect of auditory background sound on learning. This account is based on the assumption that memory improves with increasing similarity between the retrieval and the encoding context. The basic idea behind context-dependent memory is that task-irrelevant context details become automatically associated with the focal stimuli. Retrieval can benefit from the reinstatement of the encoding context because contextual details can be used as retrieval cues. For example, when the same music is played at retrieval and encoding, this could have beneficial effects on memory because the music serves as a retrieval cue^52^. However, this account does not provide a satisfactory explanation for the present findings. First, it is debatable whether the *absence* of contextual cues should benefit retrieval of faces encoded in quiet. Secondly, the encoding-retrieval match is known to have only a minor or no effect on recognition^53^. Moreover, the context dependency of learning cannot explain the main finding of the present study that changing-state sound is more disruptive than steady-state sound because both distractor conditions involve a context change between encoding and test. It also does not offer any explanation for the finding of Proverbio *et al*.^34^ that joyful music and sounds of thunder at encoding disrupted performance while touching music with smooth transitions between tones did not. Particularly troubling for the context-dependency account is that the psychophysiological measures (blood pressure and heart rate) in the Proverbio *et al*. study indicated that participants responded sensitively to the touching music that nevertheless did not have any effect on face recognition. This pattern of findings suggests that a different explanation has to be found for the disruption of face learning by irrelevant sounds.

Auditory distraction seems to provide a better explanation. Two broad classes of theories are available to explain the effects of auditory distraction on cognitive processing: The interference-by-process account (e.g.^46,54^) and the attentional account (e.g.^24^).

The first account is based on *similarity-based interference*. In general, the more similar two processes are, the more likely they are to interfere with each other^55^. The interference-by-process account of auditory distraction^46,54^ assumes that interference occurs because certain properties of the auditory distractors are automatically processed and come into conflict with processes needed to encode and to retain the to-be-remembered material. Based on the similarity-based interference principle, the finding of auditory distraction in the present paradigm is surprising given that the to-be-learned stimuli are quite dissimilar to the to-be-ignored stimuli. Not only were the faces presented in a different modality than the to-be-ignored speech, but it is also often assumed that the processing of faces requires visual-configural processing^56^ and relies on specialized processing modules in the ventral visual processing stream^57^. A priori, there is no reason to assume that similarity-based interference with background speech should occur.

To explain the present results, the interference-by-process account could invoke the auxiliary assumption that participants verbally recode the faces to encode them. The verbal recoding of faces may seem plausible when familiar faces with well-known names are used^58^, when long encoding periods are used^6^, and when the verbal description of the target face is explicitly required^59^. However, in the present study unfamiliar faces were presented briefly (700 ms with a 1200 ms inter-stimulus interval) for later visual face recognition. Furthermore, it has been shown that the verbal recoding of unfamiliar faces does not necessarily improve face-recognition performance. The verbal overshadowing effect^59,60^ refers to the observation that face recognition suffers when people are required to verbally recode a face because they have to provide a verbal description of its facial features. An explanation of this effect is that a verbal code is inappropriate for face recognition that lends itself better to non-verbalizable configural processing. According to the transfer-inappropriate processing account of the verbal overshadowing effect^61^, “verbalization induces inappropriate processing operations, which (…) are incommensurate with the processes required for successful recognition performance” (p. 992). If irrelevant speech discouraged such inappropriate verbalization attempts, this should not necessarily lead to an impairment of face recognition. Another problem of the speech-based explanation is that it cannot easily account for the finding of Proverbio *et al*.^34^ that the distraction effect generalizes to non-speech sounds. These factors make an explanation based on the interference between verbal codes unattractive. Study 2 further suggests that lexical, syntactical or semantic processing of the distractor speech can also be ruled out as causes for interference.

In the working-memory literature, the changing-state effect is often attributed to the interference between two types of order information^62^. According to this explanation, changes lead to the segmentation of the auditory stream into distinct objects, the order of which is automatically processed. The involuntary seriation of the auditory stream disrupts the voluntary seriation of to-be remembered material. An implication of this account is that the changing-state effect should only be found in tasks that rely on the processing of serial order such as serial recall^63^. In the present study, however, the order in which the faces were presented in the recognition test was unrelated to their order at encoding. It therefore seems unclear why face recognition should have relied on the processing of serial order.

By contrast, the *attentional account* explains the changing-state effect by assuming that abrupt changes in the auditory modality capture attention. Based on literature on attentional orienting^11,64–68^, the graded attentional account^24^ postulates that changes in the auditory modality elicit a call for attention. Steady-state stimuli are less disruptive than changing-state stimuli because the system adapts to repetitive stimulus patterns so that repeated steady-state distractors cause gradually less distraction^24^. Changing stimuli, by contrast, require some processing before the focal attention can be reoriented to the visual modality so that they impair performance in tasks that benefit from focal attention. Sentential speech is particularly disruptive because it contains many auditory changes^19^ that are efficiently detected due to the importance of speech processing for human communication^32^. When attention is captured by irrelevant speech, less attention is available for ongoing performance. The attentional explanation thus does not require distractors and targets to share a common representational code or to rely on the same processing modules. Instead, it assumes that less focal attention is available for modulating the domain-specific processing routines when it is involuntarily captured by auditory changes. An implication of this model is that the changing-state effect should not be strictly confined to the processing of serial order but should generalize to different kinds of encoding processes that rely on focal attention such as binding^4^ and updating^2^.

The attentional explanation of the changing-state effect on face learning thus relies on the assumption that intentional face learning is influenced by the amount of attentional resources devoted to the faces at encoding. Given the widespread belief that face processing is largely automatic, this assumption may seem surprising at first glance. However, there is substantial evidence that the processing of faces is affected by attentional load. Markers of the neuronal processing of faces such as the N170 in the event-related potential depend on selective attention^69,70^. Accordingly, there is convincing evidence that a reduction of attentional resources at encoding disrupts face learning^71^. The attentional explanation of the present results thus implies that intentional face learning is impaired when attention is captured by changes in the auditory modality. A potential implication of the attentional-diversion view is that the effect of changing irrelevant speech should be more robust in intentional face learning when participants voluntarily devote attention to the faces than when they do not attend to the faces in which case face processing may be largely automatic. Two recent studies suggest that this may be the case. When the encoding of the faces was incidental, irrelevant speech had no detectable effect on face processing relative to a quiet control condition^6,7^. By contrast, in the present study and the previous study providing evidence of changing-state disruption^34^, participants were instructed to encode the faces intentionally. The available literature thus seems to support the assumption that irrelevant speech interferes with the voluntary direction of attention to the faces rather than with the more automatic components of incidental face processing (cf.^7^).

Here we report studies showing that intentional face learning is negatively affected by irrelevant speech. This finding suggests that auditory distraction by changing-state sounds is more general and thus more relevant than previously assumed. Changing auditory distractors do not only interfere with the retention of sequences of digits (e.g., telephone numbers), but also with the learning of complex visual stimuli such as faces. This disruptive effect is not restricted to comprehensible speech as it is found with reversed speech as well. Our findings are not only relevant for the evaluation of models of auditory distraction, they are interesting from an applied perspective as well. A practical conclusion from this research is that exposure to background speech and music should be reduced when a task requires accurate face recognition (e.g., in security screenings). In eyewitness testimony, accurate recognition of the perpetrator face is crucial. Due to the lack of research, one may incorrectly assume that only visual factors—such as the view on the target, viewing distance, and light conditions—are relevant for evaluating the accuracy of eyewitness testimonies. However, the present results—along with those of two previous studies by Marsh and colleagues^6,7^—suggest that auditory distraction can have a significant detrimental effect on face-recognition accuracy. These findings thus advance our knowledge about the factors that influence face recognition in eyewitness testimony as well as in other contexts in which face recognition accuracy is of critical importance.
